# Promoting notification and linkage of HBs antigen and anti-HCV antibody-positive patients through hospital alert system

**DOI:** 10.1186/s12879-017-2438-1

**Published:** 2017-05-08

**Authors:** Naoki Yoshioka, Akihiko Okumura, Yukie Yamamoto, Katsura Yamaguchi, Atsuro Kaga, Kentaro Yamada, Takuya Hirosaki, Daisuke Ishikawa, Shin Kunii, Kazumasa Watanabe, Setsuo Utsunomiya, Kazuhiko Hayashi, Masatoshi Ishigami, Hidemi Goto, Yoshiki Hirooka

**Affiliations:** 1 Department of Gastroenterology, The Aichi Prefectural Federation of Agricultural Cooperatives for Health and Welfare Kainan Hospital, 396 Minamihonden, Maegasu-cho, Yatomi, Aichi 498-8502 Japan; 2 Department of Medical Technology, The Aichi Prefectural Federation of Agricultural Cooperatives for Health and Welfare Kainan Hospital, 396 Minamihonden, Maegasu-cho, Yatomi, Aichi 498-8502 Japan; 3 Department of Medical Oncology, The Aichi Prefectural Federation of Agricultural Cooperatives for Health and Welfare Kainan Hospital, 396 Minamihonden, Maegasu-cho, Yatomi, Aichi 498-8502 Japan; 4 Department of Gastroenterology and Hepatology, Nagoya University Graduate School of Medicine, 65 Tsurumai-cho, Showa-ku, Nagoya, 466-8550 Japan

**Keywords:** Alert system, Hepatitis B virus, Hepatitis C virus, Notification and referral

## Abstract

**Background:**

In Japan, approximately 0.9% and 1% of the whole population are infected with HBV and HCV, respectively. Doctors from departments other than gastroenterology often order viral hepatitis tests before an invasive examination or an operation. However, the notification of positive results to the patients and linkage to care is not appropriately performed. The in-hospital alert system was constructed to promote the notification and referral to gastroenterologists for patients with positive viral hepatitis tests, and its efficacy was evaluated.

**Methods:**

The patients who tested HBsAg and anti-HCV antibody by chemiluminescent enzyme immunoassays and chemiluminescent immunoassays were investigated for whether they were notified of the positive results and if they were referred to gastroenterologists at our hospital. The notification and referral rate was compared before (from January to December 2014) and after the introduction of the alert system (from February to September 2016).

**Results:**

HBsAg-positive rate was 1.1% (69/6543) before the introduction of the alert system and 0.8% (41/5403) after it. The notification rate has significantly improved from 46% to 73% (*p* = 0.0061) and the referral rate has improved from 16% to 27%, while not significant. Positive rate of anti-HCV antibody was 2.1% (139/6481) before the introduction of the alert system and 2.4% (128/5322) after it. The rate of notification and referral has significantly improved from 35% to 62% (*p* < 0.0001) and from 6% to 23% (*p* < 0.0001), respectively.

**Conclusions:**

The in-hospital alert system increased the rates of notification and referral of the patients with positive viral hepatitis tests. Enlightenment of doctors other than gastroenterologists on viral hepatitis and cooperation of medical staffs would be helpful to improve the notification and referral rates.

## Background

Viral hepatitis is a leading cause of death worldwide (1.45 million deaths in 2013) [1]. More than 90% of this burden is due to hepatitis B virus (HBV) and hepatitis C virus (HCV) as approximately 240 million individuals suffer from chronic hepatitis B, and 130–150 million suffer from chronic hepatitis C [2, 3]. Without treatment, 20–30% of HBV- and HCV-infected persons will develop hepatocellular carcinoma or cirrhosis, leading to an estimated 19 million deaths between 2015 and 2030 (11.8 million from HBV and 7.2 million from HCV) [4–6].

The World Health Organization made a commitment to eliminate viral hepatitis by 2030 at the World Health Assembly in 2016. Prevention and treatment strategies will increase the rate of treatment to 80% and reduce the number of annual deaths by 65%, saving 7.1 million lives globally by 2030 [6].

In Japan, it was estimated that there were 1.1–1.2 million HBV-infected and 1.0–1.5 million HCV-infected individuals. Tanaka et al. estimated that approximately 0.48 million HBV-infected and 0.30 million HCV-infected individuals remained unaware of their infection status in 2011 [7].

In Japan, doctors from departments other than gastroenterology often order a hepatitis B surface antigen (HBsAg) test and an anti-HCV antibody test before an invasive examination or an operation. However, the notification of positive results to the patients and linkage to care is not appropriately performed [8–10]. In this study, the rate of patient notification and gastroenterologist referral was assessed. The in-hospital alert system which promotes the notification and referral was constructed and the effects of the system were evaluated at our hospital.

## Methods

### Subjects

This was a single center study involving both inpatients and outpatients conducted at a tertiary care hospital. We searched the patients who tested HBsAg and anti-HCV antibody at departments other than the gastroenterology department through electric medical records. The records of the patients positive for HBsAg and anti-HCV antibody were examined whether they were notified the positive results, whether they were referred to gastroenterologists, and whether they received proper medical care. The rate of notification and referral was compared before (from January to December 2014) and after the introduction of the in-hospital alert system (from February to September 2016).

### The in-hospital alert system for promoting the notification of positive results and referral to gastroenterologists

The medical technologists in the laboratory sent the following comments together with the positive results of the HBsAg or anti-HCV antibody tests: “Hepatitis virus test was positive. Please print the form and explain the positive results for the hepatitis virus infection to the patient.” Within a week after the tests, the same comment was sent via e-mail to the doctors who ordered the hepatitis virus tests. Using the form, the doctors explained the positive results to the patients. After the patient had signed the form, the doctor captured it on the electronic medical records. The patients who wanted to see gastroenterologists were referred to one at our hospital. This alert system was constructed and became operational in February 2016.

### HBsAg and anti-HCV antibody tests

At the preliminary investigation, HBsAg was measured using the Lumipulse II HBsAg (Fujirebio Inc., Tokyo, Japan), and anti-HCV antibody was measured using the Lumipulse II Ortho HCV (Fujirebio Inc., Tokyo, Japan). Both assays were chemiluminescent enzyme immunoassays. Following the introduction of the alert system, HBsAg was measured using the Architect HBsAg QT assay (Abbott Japan, Tokyo, Japan) and anti-HCV antibody was measured using the Architect HCV assay (Abbott Japan, Tokyo, Japan). Both assays were chemiluminescent immunoassays.

### Statistical analysis

The statistical analysis was conducted using StatFlex ver. 6.0 software (Artech. Co., Ltd., Osaka, Japan). A chi-square test was used for categorical factors. Differences with *p* values <0.05 were considered to be statistically significant. The difference of rates was also assessed by 95% confidential interval.

## Results

### Preliminary investigation

HBsAg was measured in 6543 patients, of whom 69 (1.1%) were positive. Anti-HCV antibody was measured in 6481 patients, of whom 139 (2.1%) were positive. The major departments where the hepatitis tests were undergone were emergency department, orthopedics department, and urology department. The positive rate for anti-HCV antibody was the highest in nephrology department (4.20% [6/143]) (Table 1).

**Table 1:** Distribution of patients tested for HBsAg or anti-HCV antibody at departments other than the gastroenterology department

Department	HBsAg			anti-HCV antibody		
	Number of patients tested	Number of positive patients	Positive rate (%)	Number of patients tested	Number of positive patients	Positive rate (%)
emergency	1318	16	1.21	1391	40	2.88
orthopedics surgery	893	13	1.46	873	21	2.41
urology	759	12	1.58	754	19	2.52
gastrointestinal surgery	564	3	0.53	610	15	2.46
obstetrics and gynecology	504	3	0.60	504	1	0.20
cardiology	392	1	0.26	369	10	2.71
respiratory medicine	345	6	1.74	336	12	3.57
otorhinolaryngology	301	1	0.33	279	1	0.36
cranial nerve surgery	293	1	0.34	279	1	0.36
plastic and reconstructive surgery	242	2	0.83	251	3	1.20
ophthalmology	185	0	0	197	4	2.03
dermatology	178	2	1.12	123	2	1.63
nephrology	150	1	0.67	143	6	4.20
rheumatology	73	1	1.37	61	0	0
hematology	64	1	1.56	51	1	1.96
dental surgery	63	1	1.59	63	0	0
general medicine	57	2	3.51	50	2	4.00
neurology	49	1	2.04	48	1	2.08
pediatrics	34	1	2.94	21	0	0
diabetes medicine and endocrinology	34	0	0	31	0	0
cardiovascular surgery	29	1	3.45	29	0	0
medical oncology	6	0	0	8	0	0
geriatrics	5	0	0	4	0	0
anesthesiology	4	0	0	5	0	0
psychiatry	1	0	0	1	0	0
Total	6543	69	1.05	6481	139	2.14

Of the patients positive for HBsAg and anti-HCV antibody, 42 (61%) and 86 (62%) were male, respectively. HBsAg-positive rates were 0.4% (1/224) in teens, 0.3% (1/361) in 20’s, 0.7% (4/596) in 30’s, 0.9% (5/569) in 40’s, 2.2% (15/693) in 50’s, 1.5% (19/1267) in 60s’, 0.9% (14/1619) in 70s’, and 1.2% (10/849) in 80’s. Anti-HCV antibody-positive rates were 0.5% (3/585) in 30’s, 1.3% (7/551) in 40’s, 1.5% (10/687) in 50’s, 2.5% (32/1267) in 60’s, 2.8% (45/1615) in 70’s, 4.5% (38/850) in 80’s, and 2.9% (4/140) in 90’s.

**Fig. 1:**
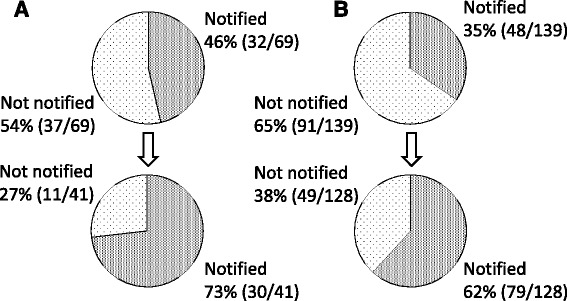
The rate of HBsAg or anti-HCV antibody-positive patients who were notified of the results before and after the introduction of the alert system. **a** The rate of HBsAg-positive patients who were notified of the positive results increased significantly after the introduction of the alert system (95% confidential interval of difference of rate = 8% ~ 46%; *p* = 0.0061). **b** The rate of anti-HCV antibody-positive patients who were notified of the results increased significantly after the introduction of the alert system (95% confidential interval of difference of rate = 15% ~ 39%; *p* < 0.0001)

**Fig. 2:**
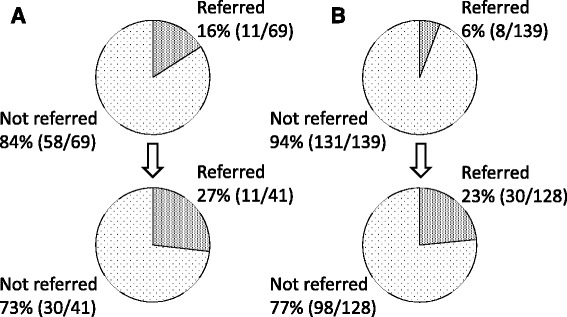
The rate of HBsAg or anti-HCV antibody-positive patients who were referred to a gastroenterologist before and after the introduction of the alert system. **a** The rate of HBsAg-positive patients who were referred to gastroenterologists increased after the introduction of the alert system, while there was no statistical significance (95% confidential interval of difference of rate = −5% ~ 26%). **b** The rate of anti-HCV antibody-positive patients who were referred to a gastroenterologist increased significantly after the introduction of the alert system (95% confidential interval of difference of rate = 9% ~ 26%; *p* < 0.0001)

A total of 46% (32/69) of the HBsAg-positive patients and 35% (48/139) of the anti-HCV antibody-positive patients were notified of the results (Fig. 1a and b). There were 16% (11/69) of the HBsAg-positive patients and 6% (8/139) of the anti-HCV antibody-positive patients who were referred to gastroenterologists (Fig. 2a and b).

### HBsAg-positive patients after the introduction of the alert system

HBsAg was measured in 5403 patients, of whom 41 (0.7%) were positive. Of the HBsAg-positive patients, 73% (30/41) were notified of the results, which was a significantly higher rate than that before the introduction of the alert system (46% [32/69]; 95% confidential interval of difference of rate = 8% ~ 46%; *p* = 0.0061) (Fig. 1a). There were 27% (11/41) of the HBsAg-positive patients who were referred to gastroenterologists, a higher rate than that before the introduction of the alert system (16% [11/69]), while there was no statistical significance (95% confidential interval of difference of rate = −5% ~ 26%) (Fig. 2a).

A total of 11 patients consulted gastroenterologists. Of these, seven patients initiated nucleoside/nucleotide analogues (NAs) therapy because of their chemotherapy or immunosuppressive therapy, three patients were diagnosed as asymptomatic carriers, and one patient declared no wish to receive an examination or treatment.

There were 19 patients who were notified of the positive results but were not referred to a gastroenterologist. Of these, three patients were not with proper care (Table 2).

**Table 2:** State of medical care of 19 patients who were notified of HBsAg-positive results but were not referred to the gastroenterologists

State of medical care	Number of patients	
With proper care	16	
Undergoing nucleoside/nucleotide analogues therapy		12
Seeing gastroenterologists at our hospital		2
Attending other general hospitals		2
Without proper care	3	
Seeing a pediatrician at our hospital		1
Seeing a general practitioner at other clinic		1
Declared no wish to receive an examination or treatment		1

### Anti-HCV antibody-positive patients after the introduction of the alert system

Anti-HCV antibodies were measured in 5322 patients, of whom 128 (2.4%) were positive. There were 62% (79/128) of the anti-HCV antibody-positive patients who were notified of the positive results, a significantly higher rate than that before the introduction of the alert system (35% [48/139]; 95% confidential interval of difference of rate = 15% ~ 39%; *p* < 0.0001)(Fig. 1b). A total of 23% (30/128) of the anti-HCV antibody-positive patients were referred to gastroenterologists, which was a significantly higher rate than that before the introduction of the alert system (6% [8/139]; 95% confidential interval of difference of rate = 9% ~ 26%; *p* < 0.0001)(Fig. 2b).

There were 30 patients who consulted gastroenterologists. Of these, three patients started interferon-free treatment; one patient began taking ursodeoxycholic acid, twenty-one patients were found to be negative for HCV RNA, and five patients declared no wish to receive an examination or treatment.

A total of 49 patients were notified of the positive results but were not referred to the gastroenterologists. Of these, 13 patients were not with proper care, seven of which declared no wish to receive an examination or treatment (Table 3). Of seven patients who declared no wish to receive an examination or treatment, one patient complained that a general practitioner told him the possible severe side effects caused by interferon-free treatment, and two patients (a 70-year-old man and an 86-year-old woman) complained that they were very old and their liver function was stable. The reason for the declaration of no wish to receive an examination or treatment in the other four patients remains unknown.

**Table 3:** State of medical care of 49 patients who were notified of anti-HCV antibody-positive results but were not referred to the gastroenterologists

State of medical care	Number of patients	
With proper care	36	
HCV RNA negative without antiviral therapy		9
Sustained virological response after antiviral therapy		13
Under interferon-free treatment		4
Seeing gastroenterologists at our hospital		6
Attending other general hospitals		4
Without proper care	13	
Seeing a surgeon at our hospital		1
Seeing general practitioners at other clinics		5
Declared no wish to receive an examination or treatment		7

## Discussion

The in-hospital alert system was constructed to promote doctors other than gastroenterologists to appropriately notify and refer patients with positive viral hepatitis tests to gastroenterologists. The system increased the rate of notification for the HBsAg (from 46 to 73%) or for anti-HCV antibody (from 35 to 62%) tests, and the rate of referral for HBsAg (from 16 to 27%) or for anti-HCV antibody (from 6% to 23%) tests.

There were only two previous reports about in-hospital alert system. Shimomura et al. reported that a fully automated reporting system on electronic medical records increased the rate of notification for patients positive for viral hepatitis tests (from 29 to 58%) [9]. Fujii et al. reported that an alert system consisting of putting “sticky notes” on the electronic medical records increased the referral rate of the HBsAg-positive patients from 14% to 48% and that of anti-HCV antibody-positive patients from 7 to 51% [10]. Thus, these alert systems are useful for increasing the number of notifications and referrals to specialists for patients with positive results of viral hepatitis test.

The referral rate of the present study was apparently lower than that of Fujii et al. We assessed the state of medical care of the patients with notification but without referral. 16% (3/19) of HBsAg-positive patients and 27% (13/49) of anti-HCV antibody-positive patients did not receive proper care, while 84 and 73% received proper care, respectively. The low rate of referral may be attributed to the high rate of proper care in those with notification but without referral.

In United States of America, Hepatitis Testing and Linkage to Care (HepTLC) initiative of the Center for Disease Control and Prevention provided financial supports to organizations to offer testing and test result notification to people at risk for HBV and/or HCV infection [11]. Of the 4766 people who tested positive for either HBV or HCV infection, 2116 (44%) were linked to care. It is suggested that patient navigation by counselors was critical for follow-up and linkage to care. In our hospital, medical counselors would be helpful to deliver the patients the information of viral hepatitis and to raise the rate of referral.

We found that the findings that some patients did not receive proper care were attributed to the inappropriate knowledge of the doctors other than gastroenterologists and patients. Thus, enlightenment of the doctors on viral hepatitis is needed. Patient navigation by medical counselor is also needed.

In the alert system, the medical technologists in the laboratory send a comment which recommends the notification and referral together with the positive results of viral hepatitis test, and send the same comment via e-mail within a week after the test results. The alert system was constructed by both doctors and medical technologists. Therefore, the medical technologists became familiar with viral hepatitis and made a greater effort to provide appropriate care to patients with viral hepatitis. To provide appropriate care to the patients, it is important to gather the support of all medical staff. The construction of the alert system provided such an opportunity to involve medical staffs other than doctors.

Today, chronic hepatitis with HBV and HCV can both be treated with highly effective medicines [12–15]. Thus, the early detection and linkage to proper care have become increasingly important [16–18]. The Ministry of Health, Labor and Welfare has implemented the recommendation to inform all Japanese citizens of the necessity of undergoing hepatitis tests at least once, and such tests are free [19]. However, the rate of tests for HBV or HCV is low (57.7 or 48.1%, respectively) [20]. Thus, it is important that when patients are found positive for HBsAg or anti-HCV antibody in the hospital, notification and referral to a gastroenterologist should be securely performed.

## Conclusions

The alert system has increased the rates of notification and referral. It is demonstrated that many of the patients positive for viral hepatitis test with notification received proper care even without referral to gastroenterologists in our hospital. However it is necessary to raise the rate of notification and referral further. The enlightenment of doctors other than gastroenterologists on viral hepatitis and cooperation of medical staffs would be helpful to raise the notification and referral rate.

## Abbreviations

HBV: hepatitis B virus
HCV: hepatitis C virus
HBsAg: hepatitis B surface antigen
SVR: sustained virological response
